# The role of angiotensin I-converting enzyme gene polymorphism and global DNA methylation in the negative associations between urine di-(2-ethylhexyl) phthalate metabolites and serum adiponectin in a young Taiwanese population

**DOI:** 10.1186/s13148-023-01502-z

**Published:** 2023-05-17

**Authors:** Chien-Yu Lin, Hui-Ling Lee, Ching-Way Chen, Chikang Wang, Fung-Chang Sung, Ta-Chen Su

**Affiliations:** 1 Department of Internal Medicine, En Chu Kong Hospital, New Taipei City, 237 Taiwan; 2 School of Medicine, Fu Jen Catholic University, New Taipei City, 242 Taiwan; 3 Department of Environmental Engineering and Health, Yuanpei University of Medical Technology, Hsinchu, 300 Taiwan; 4 Department of Chemistry, Fu Jen Catholic University, New Taipei City, 242 Taiwan; 5 Department of Cardiology, National Taiwan University Hospital Yunlin Branch, Yunlin, 640 Taiwan; 6 Department of Health Services Administration, College of Public Health, China Medical University, Taichung, 404 Taiwan; 7 Department of Environmental and Occupational Medicine, National Taiwan University Hospital, Taipei, 10002 Taiwan; 8 Department of Internal Medicine and Cardiovascular Center, National Taiwan University Hospital, Taipei, 100 Taiwan; 9 Institute of Environmental and Occupational Health Sciences, College of Public Health, National Taiwan University, Taipei, 100 Taiwan; 10 The Experimental Forest, National Taiwan University, Nantou, 558 Taiwan

**Keywords:** 5mdC/dG, Adiponectin, DEHP (di-(2-ethylhexyl) phthalate), Epigenetic modifications, Glucose homeostasis, MEHP (mono(2-ethylhexyl) phthalate)

## Abstract

**Background:**

Adiponectin is a key protein produced in adipose tissue, with crucial involvement in multiple metabolic processes. Di-(2-ethylhexyl) phthalate (DEHP), one of the phthalate compounds used as a plasticizer, has been shown to decrease adiponectin levels in vitro and in vivo studies. However, the role of angiotensin I-converting enzyme (ACE) gene polymorphism and epigenetic changes in the relationship between DEHP exposure and adiponectin levels is not well understood.

**Methods:**

This study examined the correlation between urine levels of DEHP metabolite, epigenetic marker 5mdC/dG, ACE gene phenotypes, and adiponectin levels in a sample of 699 individuals aged 12–30 from Taiwan.

**Results:**

Results showed a positive relationship between mono-2-ethylhexyl phthalate (MEHP) and 5mdC/dG, and a negative association between both MEHP and 5mdC/dG with adiponectin. The study found that the inverse relationship between MEHP and adiponectin was stronger when levels of 5mdC/dG were above the median. This was supported by differential unstandardized regression coefficients (− 0.095 vs. − 0.049, *P* value for interaction = 0.038)). Subgroup analysis also showed a negative correlation between MEHP and adiponectin in individuals with the I/I ACE genotype, but not in those with other genotypes, although the *P* value for interaction was borderline significant (0.06). The structural equation model analysis indicated that MEHP has a direct inverse effect on adiponectin and an indirect effect via 5mdC/dG.

**Conclusions:**

In this young Taiwanese population, our findings suggest that urine MEHP levels are negatively correlated with serum adiponectin levels, and epigenetic modifications may play a role in this association. Further study is needed to validate these results and determine causality.

**Supplementary Information:**

The online version contains supplementary material available at 10.1186/s13148-023-01502-z.

## Background

Adiponectin is a protein consisting of 244 amino acids that is produced by fat cells. It plays a role in regulating various metabolic processes, including improving insulin sensitivity. [1]. Adiponectin has been reported to play protective roles against diabetes mellitus, cancer, and cardiovascular disease (CVD) [2]. A number of factors can influence the amount of adiponectin present in the body, such as peroxisome proliferator-activated receptor (PPAR) *γ*, insulin, insulin-like growth factor, leptin, and various inflammatory cytokines [3, 4]. The renin–angiotensin–aldosterone system (RAAS), a series of peptide hormones that regulates body fluid, also plays a role in regulating adiponectin homeostasis [4, 5]. It has been shown that aldosterone may downregulate the gene expression of adiponectin [6]. Additionally, research has revealed that epigenetic modifications, which do not involve changes to the DNA sequence but can affect gene expression, can alter the expression of adiponectin-related genes [7, 8].

Di-(2-ethylhexyl) phthalate (DEHP), a commonly used plasticizer, is a type of phthalate compound that can be easily released into the ecosystem due to its lack of covalent bonding with plastics [9]. DEHP exposure has been linked to various adverse health effects, such as endocrine and metabolic diseases [9]. According to experimental studies, DEHP treatment may reduce adiponectin levels [10–12]. The exact mechanism through which DEHP leads to a decrease in adiponectin concentration is still being studied. Possible mechanisms include decreasing expression of PPARγ, increasing inflammatory cytokines and oxidative stress [10–12].

Animal studies have also shown that DEHP exposure is linked to the stimulation of the RAAS. Possible mechanisms for this effect include the elevation of angiotensin I-converting enzyme (ACE) levels and the suppression of 11β hydroxysteroid dehydrogenase type 2 (11β-HSD-2) enzyme activity [13, 14]. ACE is an enzyme that cleaves angiotensin 1 into angiotensin II and inactivates bradykinin [15]. It has been observed that different ACE genotypes may be associated with varying risks for CVD [15, 16]. While we know that DEHP can affect the RAAS and that RAAS can in turn influence adiponectin secretion, it is currently unclear what role ACE genotypes may play in the relationship between DEHP exposure and adiponectin levels.

Studies have also demonstrated that DEHP exposure can lead to epigenetic regulation in experimental settings [17, 18]. Past research has suggested that DNA methylation, a well-studied form of epigenetic regulation, may be involved in the link between DEHP and health outcomes [19, 20]. Although it is known that epigenetic modifications can also affect adiponectin levels [7, 8], the potential impact of these changes on the relationship between DEHP and adiponectin has yet to be explored.

Epidemiological studies exploring the correlation between DEHP exposure and adiponectin levels are limited, and the findings are conflicting. While some research has identified a positive correlation between DEHP exposure and adiponectin levels [21, 22], others have found a negative relationship [23]. Furthermore, these studies have primarily focused on children, women, and patients with diabetes mellitus, leaving a gap in knowledge about the effects in adolescents and young adults. In an effort to address this gap and better understand the relationship between DEHP exposure, serum adiponectin levels, and ACE gene polymorphism/epigenetic modification, we conducted a cross-sectional study among Taiwanese adolescents and young adults using 5mdC/dG, a marker of global DNA methylation, as a marker of epigenetic modification. The aim of this study was to examine the relationship between DEHP exposure and serum adiponectin levels in young people, as well as the role that ACE gene polymorphism/epigenetic modification may play in this association.

## Materials and methods

### Study population and data collection

Between 2006 and 2008, we conducted a cohort study (YOTA, Young TAiwanese Cohort Study) of 886 young Taiwanese individuals (aged 12–30) selected from a nationwide urine screening program [24]. The study received approval from the National Taiwan University Hospital Research Ethics Committee, and participants were enrolled after providing informed consent. We excluded 17 individuals with diabetes because their medications could potentially affect adiponectin levels [25]. Another 17 participants were excluded due to a lack of data on urine DEHP metabolites, and an additional 153 subjects were excluded due to a lack of data on global DNA methylation or serum adiponectin. A total of 699 participants were included in the final analysis. More detailed information can be found in Additional file 1.

### Anthropometric and biochemical data

In this study, we conducted a comprehensive analysis of various demographic, lifestyle, and medical factors among a group of study participants. We collected data on age, gender, household income, smoking status, alcohol consumption, and past medical history, including hypertension and diabetes mellitus. We also measured body measures such as the *z*-score of body mass index (BMI) and systolic blood pressure (SBP). Blood samples were taken from all participants after an overnight fast, and commercially available kits were used to measure serum levels of insulin (Immulite 2000; RRID: AB_2750939; Siemens Healthcare Diagnostics;) and adiponectin (Human Adiponectin/Acrp30 Immunoassay; RRID: AB_2783020; R&D Systems, Minneapolis, MN). In addition, we measured a range of other biochemistry parameters, including low-density lipoprotein cholesterol (LDL-C), high-density lipoprotein cholesterol (HDL-C), triglycerides, and the homeostasis model assessment of insulin resistance index (HOMA-IR) and β-cell function (HOMA-β), were also measured. The complete details of our methods are provided in Additional file 1.

### Measurements of urine DEHP concentrations

Once inside the human body, DEHP is catalyzed into MEHP by lipases and esterases. A range of byproducts are subsequently generated, including mono(ethyl-5-hydroxyhexyl) phthalate (MEHHP) and mono(2-ethly-5-oxoheyl) phthalate (MEOHP)). Our previous research study introduced the detailed methods [26]. The lower limit of detection (LOD) for this study was 0.5 ng/mL. For concentrations that fell below this threshold, we used a value equal to the LOD divided by the square root of 2 in our analyses. Additional information on this method can be found in Additional file 1.

### Analysis of leukocyte global DNA methylation levels

In this study, we measured 5mdC/dG and adjusted for the corresponding 15N-labeled internal standards. The detailed method for this measurement has been previously described in our studies [27]. Additional information on this method can be found in Additional file 1.

### Genotyping of ACE gene alleles

DNA was extracted from peripheral leukocytes of volunteers using saline–EDTA extraction. Genotyping of the ACE alleles (D/D, I/D, I/I) was conducted using real-time polymerase chain reaction with fluorescently labeled primers [28, 29]. The complete details are provided in Additional file 1: Methods section.

### Statistical analysis

DEHP metabolites, adiponectin, and 5mdC/dG were expressed as geometric means with standard error, and their levels were compared using Student's two-tailed *t*-test or one-way analysis of variance in different subgroups. We also examined the relationship between and DEHP metabolites, adiponectin, 5mdC/dG, and markers of glucose homeostasis using multiple linear regression analysis. The model was adjusted for age, gender, BMI *z* score, smoking status, drinking status, and family income. Due to deviation from normal distribution in the levels of DEHP metabolites, 5mdC/dG, and adiponectin, we employed a natural logarithm transformation (ln) for these three values. In the analysis, unstandardized regression coefficients were utilized to indicate the degree of change in the dependent variable that is related to a one-unit alteration in the independent variable. To evaluate the dose response relationship, DEHP metabolites were also stratified across the population into quartiles. To gain further insights, we analyzed the data to examine the connection between ln-adiponectin and ln-MEHP levels at different levels of 5mdC/dG (cut at the 50th percentile) and at different ACE genotypes. We calculated cross product terms to estimate the interaction in these analyses. We also used a structural equation model (SEM) to explore the relationships between MEHP, 5mdC/dG, and adiponectin. In the SEM, we posited that adiponectin was modified by MEHP both directly and indirectly through 5mdC/dG, and used the same covariates as in the multiple linear regression analysis. We analyzed the data using IBM SPSS Amos version 23, and considered a *P*-value of less than 0.05 to be statistically significant.

## Results

In this study, 286 male and 413 female participants aged 21.45 years old (between the ages of 12 and 30) were included. The detection rate of urine DEHP metabolites was 78.4%. The geometric means (S.E.) of the DEHP metabolites, adiponectin, and 5mdC/dG in the different subgroups are presented in Table 1. Urine MEHP concentrations were found to be higher among participants aged 12–19, whereas urine MEOHP levels were elevated in females and individuals with lower BMI *z* scores. Serum adiponectin levels were higher in females, lower BMI *z* scores, and subjects without hypertension. Higher 5mdC/dG percentage was found in subjects with current alcohol consumption and higher BMI *z* scores. In the other subgroups, no significant association was found. Multiple linear regression models were used to examine the relationship between CVD risk factors and a one-unit increase in natural ln-DEHP metabolites, ln-adiponectin, and ln-5mdC/dG. The results are presented in Additional file 1: Table S1. A one-unit increase in ln-MEHP and ln-5mdC/dG was positively correlated with BMI *z* scores. Ln-adiponectin levels were negatively associated with BMI *z* scores and LDL-C, while positively associated with HDL-C. The mean of CVD risk factors across ACE gene alleles is demonstrated in Additional file 1: Table S2. Between CVD risk factors and ACE gene alleles, no significant associations were found.

**Table 1:** Geometric means (S.E.) of the urine DEHP metabolites, serum adiponectin, and 5mdC/dG in different subgroups

	n	MEHP (μg/g creatinine)	MEHHP (μg/g creatinine)	MEOHP (μg/g creatinine)	n	Adiponectin (ng/mL)	n	5mdC/dG (%)
Overall Age (year)	699	5.12 (1.10)	27.37 (1.04)	16.87 (1.04)	450	6484.05 (1.05)	626	2.11 (1.02)
12–19	196	9.02 (1.17)^**^	27.09 (1.08)	16.12 (1.08)	92	5675.42 (1.13)	177	2.06(1.05)
20–39	503	4.10 (1.13)^**^	27.49 (1.04)	17.17 (1.04)	358	6710.31 (1.06)	449	2.62 (1.03)
*Gender*								
Male	286	4.22 (1.17)	25.73 (1.06)	15.25 (1.06)^*^	193	5460.52 (1.07)^*^	255	2.14 (1.04)
Female	413	5.85 (1.13)	25.56 (1.05)	18.09 (1.05)^*^	257	7377.57 (1.07)^*^	371	2.09 (1.03)
*Household income (TWD)*								
< 50,000 per month	266	4.15 (1.18)	26.09 (1.07)	15.97 (1.07)	178	7215.60 (1.09)	227	2.10 (1.04)
≥ 50,000 per month	432	5.81 (1.13)	28.22 (1.04)	17.47 (1.04)	272	6046.29 (1.06)	398	2.11 (1.03)
*Smoking status*								
Not active smokers	578	5.51 (1.11)	27.91 (1.04)	17.22 (1.04)	371	6658.17 (1.06)	515	2.12 (1.03)
Active smokers	121	3.61 (1.28)	24.96 (1.10)	15.29 (1.09)	79	5726.73 (1.12)	111	2.08 (1.06)
*Current alcohol consumption*								
No	635	4.92 (1.11)	27.52 (1.04)	16.98 (1.04)	411	6506.78 (1.05)	569	2.07 (1.03)^*^
Yes	63	7.54 (1.35)	26.12 (1.10)	15.89 (1.10)	39	6248.52 (1.19)	56	2.50 (1.08)^*^
*BMI z score (kg/m*^*2*^*)*								
< − 0.212	348	4.66 (1.15)	29.34 (1.05)	18.47 (1.06)^*^	218	8787.99 (1.08)^**^	316	2.00 (1.04)^*^
≥ − 0.212	351	5.62 (1.15)	25.55 (1.05)	15.43 (1.05)^*^	232	4874.62 (1.06)^**^	310	2.23 (1.03)^*^
*Hypertension*								
No	646	4.96 (1.11)	27.55 (1.40)	17.00 (1.04)	407	6756.77 (1.05)^*^	581	2.11 (1.03)
Yes	53	7.57 (1.47)	25.34 (1.15)	15.41 (1.15)	43	4391.39 (1.17)^*^	45	2.13 (1.10)
*ACE genotypes*								
D/D	73	6.04 (1.36)	31.01 (1.12)	18.70 (1.12)	52	5947.94 (1.18)	62	2.28 (1.08)
I/D	292	5.30 (1.16)	27.42 (1.06)	16.94 (1.06)	183	6122.95 (1.07)	263	2.14 (1.04)
I/I	333	4.75 (1.15)	26.57 (1.05)	16.42 (1.05)	214	6999.54 (1.08)	301	2.04 (1.03)

Tests by Student’s two-tailed *t*-test or one-way analysis of variance. *P < 0.05 **P < 0.001

*ACE* Angiotensin-converting enzyme; *DEHP* Di-(2-ethylhexyl) phthalate; *MEHP* Mono(2-ethylhexyl) phthalate; *MEHHP* Mono(2-ethyl-5-hydroxyhexyl) phthalate; *MEOHP* Mono(2-ethyl-5-oxohexyl) phthalate; *TWD* New Taiwan dollar

Multiple linear regression models were used to examine the relationship between markers of glucose homeostasis, adiponectin, and 5mdC/dG and a one-unit increase in ln-urine phthalates metabolites, adiponectin, and 5mdC/dG. The results are presented in Table 2. Our findings indicate that MEHP concentrations negatively affect adiponectin levels and positively influence insulin, HOMA-IR, HOMA-β, and 5mdC/dG. Adiponectin levels were inversely related to insulin, HOMA-IR, HOMA-β, and 5mdC/dG while 5mdC/dG was positively associated with insulin, HOMA-IR, HOMA-β, and negatively linked to adiponectin. The geometric mean of adiponectin and 5mdC/dG levels across quartiles of MEHP levels is demonstrated in Table 3. The mean levels of adiponectin significantly decreased with increasing quartiles of MEHP (*P* for trend < 0.001) while the mean 5mdC/dG percentage rose with quartiles of MEHP (*P* for trend < 0.001).

**Table 2:** Unstandardized regression coefficients (standard error) of markers of glucose homeostasis, ln-adiponectin, and ln-5mdC/dG with a one-unit increase in ln-urine DEHP metabolites, ln-adiponectin, and ln-5mdC/dG in multiple linear regression models

	N		DEHP metabolites (μg/g creatinine)			Ln-5mdC/dG (%)	Ln-Adiponectin (ng/mL)
			MEHP	MEHHP	MEOHP		
Fasting glucose (mg/dL)	698	Adjusted *β* (SE)	0.024 (0.092)	0.009 (0.237)	− 0.052 (0.244)	0.258 (0.414)	0.140 (0.308)
		*P* value	0.797	0.969	0.830	0.534	0.649
Ln-Insulin (uIU/mL)	698	Adjusted *β* (SE)	0.047 (0.012)	0.006 (0.032)	0.010 (0.033)	0.138 (0.056)	− 0.102 (0.038)
		*P* value	< 0.001	0.852	0.766	0.013	0.008
Ln-HOMA-IR	698	Adjusted *β* (SE)	0.048 (0.013)	0.006 (0.033)	0.009 (0.033)	0.141 (0.057)	− 0.100 (0.039)
		*P* value	< 0.001	0.853	0.783	0.014	0.011
Ln- HOMA-β	698	Adjusted *β* (SE)	0.046 (0.012)	0.005 (0.031)	0.010 (0.032)	0.133 (0.054)	− 0.107 (0.038)
		*P* value	< 0.001	0.875	0.744	0.013	0.005
Ln-Adiponectin (ng/ml)	439	Adjusted *β* (SE)	− 0.079 (0.019)	0.012 (0.051)	0.018 (0.051)	− 0.219 (0.086)	
		*P* value	< 0.001	0.820	0.730	0.011	
Ln-5mdC/dG (%)	612	Adjusted *β* (SE)	0.051 (0.010)	0.036 (0.025)	0.046 (0.026)		− 0.085 (0.033)
		*P* value	< 0.001	0.152	0.073		0.011

Model adjusted for age, gender, BMI *z* score, smoking status, drinking status, household income

**Table 3:** Geometric mean (SE) of adiponectin and 5mdC/dG across quartiles of MEHP in multiple linear regression models

	Adiponectin (ng/ml)			5mdC/dG (%)		
	Geometric mean (SE)	*P* value	*P* for trend	Geometric mean (SE)	*P* value	*P* for trend
MEHP (μg/g creatinine)			< 0.001			< 0.001
1st quartile (< 1.92)	8510.02 (1.12)	Reference		1.93 (1.06)	Reference	
2nd quartile (< 12.94)	7051.53 (1.12)	0.781		1.92 (1.06)	1.000	
3rd quartile (< 38.10)	6222.95 (1.13)	0.091		2.48 (1.06)	0.001	
4th quartile (≥ 38.10)	4230.18 (1.01)	< 0.001		2.88 (1.06)	< 0.001	

Model adjusted for age, gender, BMI *z* score, smoking status, drinking status, household income

**Fig. 1:**
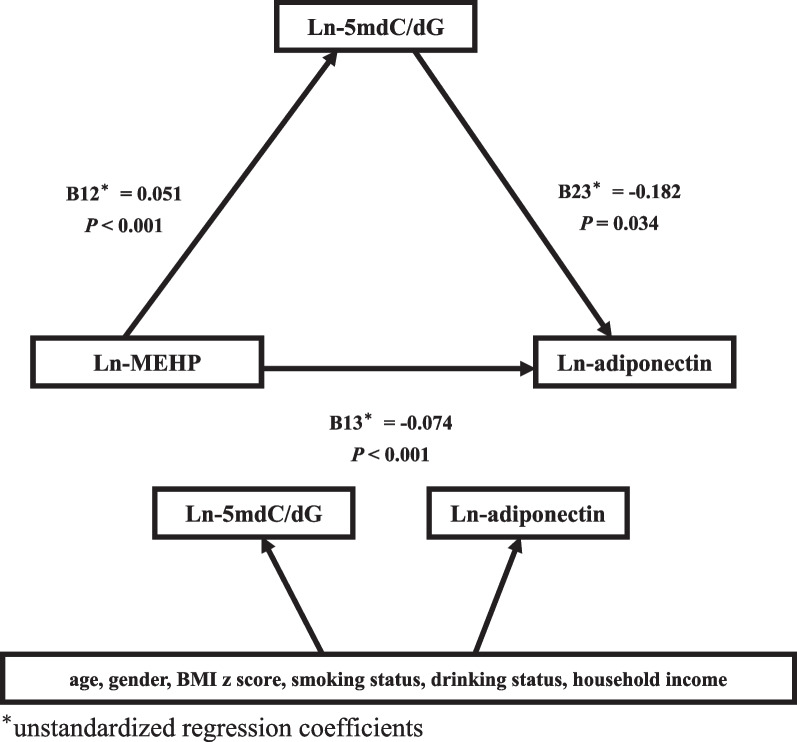
Relationship between MEHP, 5mdC/dG, and adiponectin in the structural equation model. *Unstandardized regression coefficients

Table 4 shows the unstandardized regression coefficients of the ln-adiponection with a one-unit increase in the ln-MEHP level for different categories of the 5mdC/dG levels and ACE gene categories. The study found that when the 5mdC/dG level was above the 50th percentile (Adjusted β (SE), *P* value = 0.002), the ln-MEHP level was negatively correlated with adiponectin. Additionally, when the 5mdC/dG level was above the 50th percentile, the unstandardized regression coefficient was lower than below the 50th percentile (− 0.095 vs. -0.049) and a statistically significant *P* value of interaction (0.038). MEHP levels were found to be negatively correlated with adiponectin levels when the ACE genotype was I/I. However, the *P* value for this interaction was borderline significant (0.060). The SEM for MEHP, 5mdC/dG, and adiponectin is shown in Fig. 1. It was discovered that ln-MEHP levels were positively correlated with ln-5mdC/dG levels, and negatively correlated with ln-adiponectin levels. Additionally, ln-5mdC/dG levels were found to be negatively correlated with ln-adiponectin levels. The goodness of fit index, which was greater than 0.9, the normed fit index, which was also greater than 0.9, and the root mean square, which was lower than 0.05, all indicated that the model fit well.

**Table 4:** Unstandardized regression coefficients (standard error) of ln-adiponectin levels with a one-unit increase in ln-MEHP concentration by different categories of 5mdC/dG and ACE gene polymorphism and their cross product in analysis

	No	Adjusted *β* (SE)	*P* value	*P* for interaction
5mdC/dG				0.038
Total	353	− 0.078 (0.021)	< 0.001	
5mdC/dG ≤ 50th percentile	176	− 0.049 (0.030)	0.100	
5mdC/dG > 50th percentile	177	− 0.095 (0.031)	0.002	
ACE gene phenotype				0.060
Total	438	− 0.078 (0.019)	< 0.001	
D/D	52	− 0.128 (0.063)	0.068	
I/D	179	− 0.021 (0.027)	0.435	
I/I	207	− 0.098 (0.028)	< 0.001	

Model adjusted for age, gender, BMI *z* score, smoking status, drinking status, household income

## Discussion

In this study, we found that MEHP concentrations were negatively correlated with adiponectin and positively correlated with markers of glucose homeostasis, including insulin, HOMA-IR, and HOMA-β. We also discovered that MEHP had a direct negative association with adiponectin and an indirect negative impact on adiponectin via its relationship with 5mdC/dG in the SEM. In subgroup analysis, we observed a negative correlation between MEHP and adiponectin in individuals with the I/I ACE genotype, but not in others, although the *P* value for this interaction was borderline significant. This study is the first to examine the relationship between DEHP exposure, epigenetic modifications, ACE gene polymorphism, and adiponectin levels. Our findings suggest that epigenetic changes may be involved in the mechanism of DEHP-induced decreases in adiponectin concentrations.

Increased levels of MEHP in urine were found to be associated with indicators of glucose regulation, including insulin, HOMA-IR, and HOMA-β in this study. The balance between increased insulin resistance and β-cell function has no impact on blood glucose. Earlier research has demonstrated that DEHP exposure can alter glucose homeostasis in animal models [30, 31]. Some epidemiological studies have identified a relationship between DEHP exposure and insulin resistance and the prevalence of diabetes mellitus [32, 33]. In contrast, a different study did not find a relationship in an elderly population [34]. The differences in these studies might be a result of the diverse demographics of the study participants.

Several endocrine disruptors, such as bisphenol A and polyfluoroalkyl substances, have been associated with adiponectin levels [35, 36]. DEHP treatment has been shown to decrease adiponectin levels in experimental studies. In a human cell culture model, a low dose of DEHP resulted in decreased adiponectin levels and increased generation of oxygen-derived free radicals in the supernatant of treated adipocytes [10]. In rats, high-dose DEHP led to the secretion of inflammatory cytokines, disturbed lipid metabolism, and decreased adiponectin levels [11]. Additionally, high-dose DEHP has been reported to impair fertility and decrease the expression of PPARγ mRNA and adiponectin in female mice [12]. Besides mechanisms mentioned above, DEHP exposure may activate RAAS, in which aldosterone may downregulate serum levels of adiponectin [6]. The mechanisms by which DEHP affects RAAS include increasing ACE levels [13], inhibiting 11β-HSD-2 activity [14]. Furthermore, DEHP exposure could potentially increase the likelihood of obesity [37], and adipocytes are capable of producing and releasing aldosterone, which has direct negative effect on adiponectin [6, 38]. Previous research has indicated that inhibiting the RAAS can boost adiponectin levels in individuals with metabolic syndrome, and also improves expression of adiponectin in human preadipocyte cell lines [4].

The RAAS is a hormone system that is crucial for maintaining cardiovascular homeostasis and electrolyte balance. One of the components of RAAS is ACE, which is a type of enzyme called a zinc metallopeptidase. The main function of ACE is to convert angiotensin 1 into angiotensin II, a hormone that constricts blood vessels and elevates blood pressure. ACE also inactivates bradykinin, a molecule that promotes the dilation of blood vessels and lowers blood pressure. [15]. The ACE gene is located on chromosome 17q23 and is made up of 21 kilobases of DNA. The gene contains a polymorphism in the sequence of its intron 16. There are three genotypes that result from this polymorphism: those with two copies of the insertion (I/I), those with one copy of the insertion and one copy of the deletion (I/D), and those with two copies of the deletion (D/D). Individuals with the DD genotype have the highest serum ACE levels, followed by those with the I/D genotype, and finally those with the I/I genotype [39]. The ID and DD genotypes have been linked to a greater risk of CVD in several studies [15, 16]. While previous studies have looked at the relationship between DEHP exposure and adiponectin, none have specifically explored the role of ACE polymorphism in this association. Our study found that the inverse relationship between MEHP and adiponectin was only evident in individuals with the I/I ACE genotype. However, the *P*-value for interaction was only borderline significant, and there are two possible explanations for these results. Firstly, the ACE genotype may modify the negative association between MEHP and adiponectin levels, and the insignificant *P*-value for interaction might be due to inadequate power or sample size to detect a significant effect. Specifically, the effect of MEHP on adiponectin concentration may be more pronounced in individuals with the I/I genotype who have low baseline ACE levels. Additionally, MEHP might influence the expression of the ACE gene through epigenetic mechanisms. Secondly, it is also possible that the significant correlation observed in the I/I genotype group is due to chance or random error, while the lack of correlation in the other genotype groups is simply due to data variability. There is no evidence of differential effects by ACE genotype. In summary, further research with larger sample sizes is necessary to confirm the results of our study.

Gene expression can be modified by epigenetic changes, which do not alter the DNA sequence [8]. The attachment of methyl groups to cytosine rings within guanine residues, a process called DNA methylation, is the most extensively investigated epigenetic modification due to its relative ease of measurement [7]. DNA methylation has been shown to predict the likelihood of developing diabetes, even when traditional risk factors are taken into account. It is also thought to be involved in the biological pathways connecting traditional risk factors to diabetes [40]. In this study, we found that higher levels of 5mdC/dG were associated with higher insulin levels, HOMA-IR, and β cell function. This aligns with recent research suggesting that changes in DNA methylation may impact glucose homeostasis [40].

We observed that lower levels of 5mdC/dG were related to higher levels of serum adiponectin. Previous studies have shown that epigenetic modifications can affect genes expression related to adiponectin [7, 8]. A Japanese study involving 232 pairs of monozygotic twins conducted an epigenetic variation association study to identify epigenetic regulators influencing adiponectin levels. The researchers found that methylation levels at 38 specific CpG sites were consistently linked to adiponectin levels. Additionally, they discovered that epigenetic modifications affecting adiponectin may be influenced by genes with diverse functions, such as glucose and lipid metabolism, rather than genes with a specific function [8]. Given that there are numerous genes that influence adiponectin levels, it is likely that global DNA methylation markers would be found to be related to adiponectin levels in this study.

The exact way underlying DEHP's disruption of DNA methylation is not yet understood. However, previous studies have shown that MEHP can cause oxidative DNA damage in human tissue cultures [41, 42]. DNA damage caused by oxidative stress may potentially disrupt proteins that bind to methylated cytosine and guanine residues and alter the function of DNA methyltransferases, leading to epigenetic modifications [43]. A majority of the studies examining the relationship between DEHP exposure, DNA methylation, and health outcomes have centered on investigating the impact on fertility and child development [44, 45] or atherosclerosis [19, 20]. Given the lack of understanding about the role of epigenetic modifications in the development of DEHP-related health issues, this study is the first to suggest that DNA methylation may be involved in the link between DEHP exposure and adiponectin levels.

Epidemiological study reports on the relationship between DEHP exposure and serum adiponectin levels are scarce and conflicting. One study showed the total levels of DEHP metabolites in urine (∑DEHP) were positively associated with serum adiponectin levels in 459 Korean women of reproductive age [22]. Furthermore, a study involving 167 Japanese mother–child pairs examined the relationship between maternal MEHP levels and cord blood adiponectin. The results showed that maternal blood MEHP levels measured during the third trimester were positively associated with cord blood adiponectin levels in boys [21]. On the other hand, a study found that urine levels of ∑DEHP were positively associated with tumor necrosis factor-α and negatively associated with serum adiponectin levels in subgroup of 329 Chinese adults who were diagnosed with diabetes mellitus and had higher BMI [23]. In our current study, we found that there is a negative correlation between urine levels of MEHP and serum adiponectin levels in a young population. The varied ethnicity, age, and gender of the research subjects may be the main reasons for the differing results.

The limitations of this study include the lack of causal inference in a cross-sectional cohort, the limited study population consisting of young Taiwanese individuals, the lack of consideration for other environmental pollutants, and the absence of measurements of methylation levels at specific gene loci.

## Conclusions

In the YOTA cohort, we observed a positive correlation between urine levels of MEHP and 5mdC/dG and markers of glucose homeostasis, as well as a negative correlation between MEHP and serum adiponectin levels. Additionally, we found that there is a direct negative relationship between MEHP and adiponectin, meaning that higher levels of MEHP are associated with lower levels of adiponectin. We also observed an indirect negative association between MEHP and adiponectin through 5mdC/dG. These findings suggest that epigenetic changes may play a role in the pathological mechanism by which DEHP reduces adiponectin production. Our results provide new insight into the ways in which DEHP affects serum adiponectin levels and highlight the need for further research to better understand the health effects of DEHP.

## Supplementary Information

**Additional file 1.** Materials and Methods and Tables.

## Data Availability

The raw datasets used in the current study are available from the corresponding author and with permission of National Taiwan University Hospital on reasonable request.

## Abbreviations

11β-HSD-2: 11β Hydroxysteroid dehydrogenase type 2
ACE: Angiotensin I-converting enzyme
BMI: Body mass index
CVD: Cardiovascular disease
DEHP: Di-(2-ethylhexyl) phthalate
HDL-C: High-density lipoprotein cholesterol
HOMA-IR: Homeostasis model assessment of insulin resistance index
HOMA-β: Homeostasis model assessment of β-cell function
LOD: Lower limit of detection
LDL-C: Low-density lipoprotein cholesterol
PPAR: Peroxisome proliferator-activated receptor
MEHHP: Mono(ethyl-5-hydroxyhexyl) phthalate
MEOHP: Mono(2-ethly-5-oxoheyl) phthalate
RAAS: Renin–angiotensin–aldosterone system
SEM: Structural equation model
SBP: Systolic blood pressure
YOTA: Young TAiwanese Cohort Study
