# Supplementary material for: The role of angiotensin I-converting enzyme gene polymorphism and global DNA methylation in the negative associations between urine di-(2-ethylhexyl) phthalate metabolites and serum adiponectin in a young Taiwanese population
Source: Clin Epigenetics. 2023 May 17;15:87. doi: 10.1186/s13148-023-01502-z (PMC10189977; doi:10.1186/s13148-023-01502-z)
Supplement: Supplementary file 1 — Additional file 1. Materials and Methods and Tables. [file 13148_2023_1502_MOESM1_ESM.docx]

**The role of angiotensin I-converting enzyme gene polymorphism and global DNA methylation in the negative associations between urine di-(2-ethylhexyl) phthalate metabolites and serum adiponectin in a young Taiwanese population**

Chien-Yu Lin,^1,2,3^ Hui-Ling Lee,^4^ Ching-Way Chen,^5^ Chikang Wang,^3^ Fung-Chang Sung,^6^ and Ta-Chen Su^7, 8, 9, 10*^

^1^ Department of Internal Medicine, En Chu Kong Hospital, New Taipei City 237, Taiwan

^2^ School of Medicine, Fu Jen Catholic University, New Taipei City 242, Taiwan

^3^ Department of Environmental Engineering and Health, Yuanpei University of Medical Technology, Hsinchu 300, Taiwan

^4^ Department of Chemistry, Fu Jen Catholic University, New Taipei City 242, Taiwan

^5^ Department of Cardiology, National Taiwan University Hospital Yunlin Branch, Yunlin 640, Taiwan

^6^Department of Health Services Administration, College of Public Health, China Medical University, Taichung 404, Taiwan

^7^ Department of Environmental and Occupational Medicine, National Taiwan University Hospital, Taipei 10002, Taiwan

^8^ Department of Internal Medicine and Cardiovascular Center, National Taiwan University Hospital, Taipei 10002, Taiwan

^9^ Institute of Environmental and Occupational Health Sciences, College of Public Health, National Taiwan University, Taipei, 10002, Taiwan

^10^ The Experimental Forest, National Taiwan University, Nantou, 558, Taiwan

**Supplementary material**

**Materials and methods**

***Study population and data collection***

From 1992 to 2000, approximately 2,615,000 to 2,932,000 school-age children in grades 1 to 12 received an annual urine screening of urine strip by the Chinese Foundation of Health in Taipei, Taiwan. Subjects with abnormal results from two tests for proteinuria, glycosuria, or hematuria underwent a third urine screening test and a general health check-up. A total of 103,756 school children received the health check-ups and the third urine screen. Among these children, 9,227 had elevated blood pressure (EBP) and 94,529 had normal BP based on the American Heart Association criteria (2004).

From 2006 to 2008 we established a cohort, the YOung TAiwanese Cohort (YOTA) study, based on students with and without childhood EBP, selected from the 1992–2000 urine mass screening population. In the follow-up, we mailed invitation letters to eligible students in the Taipei area. After 3–5 days, 12 trained assistants and nurses conducted telephone interviews inviting those subjects with childhood EBP to come in for a follow-up health examination. No telephone interview contact was made with normotensive students. Among the 707 subjects with EBP in childhood, 303 completed the follow-up health examinations, giving a response rate of 42.9%. Among the 6,390 subjects with normal BP in childhood, 486 completed the follow-up health examinations, giving a response rate of 7.6%. In order to differentiate the effects of environment on age of exposure, we recruited 97 subjects as “best friend controls” in the cohort follow-up period. A total of 886 subjects were included in this study. Physical check-ups were given after written informed consent (NTUH). All methods in this study were performed in accordance with the relevant guidelines and approved by the Research Ethics Committee of at the National Taiwan University Hospital (NTUH). Among these subjects, we excluded 17 participants with diabetes because the medications they were taking could potentially affect adiponectin levels[1]. Another 17 participants were excluded due to lack of measurement of urine DEHP metabolites. An additional 153 subjects were eliminated because they had no data for global DNA methylation marker or serum adiponectin. Finally, there were 699 participants selected at the final analysis.

***Anthropometric and biochemical data***

Demographic data were collected during the interview. Household income was categorized as above or below 50,000 New Taiwan Dollars (NTD) per month. Alcohol consumption was divided into current alcohol consumption or not. Smoking status was divided into not active smokers and active smokers. It would be misleading to use body mass index (BMI) for adults to interpret the BMI of children and adolescents because there are changes in weight and height with age, as well as their relation to body fatness. For children and adolescents, BMI is age- and sex-specific and is often referred to as BMI-for-age and expressed as a percentile. BMI index z-scores are measures of relative weight adjusted for child age and sex. The BMI was calculated as weight in kilograms divided by height in meters squared. For participations ≥ 20 year-old, BMI z-score was measured by equation (BMI of each participants - mean of BMI)/(standard deviation of BMI) while aged 12-19 years were calculated based on WHO anthropometric calculator [2]. Blood pressure was measured twice after 3 min of rest using a mercury manometer. In adults, hypertension was defined as the self-reported current use of anti-hypertensive medication or either average systolic blood pressure ≥ 140 mmHg or average diastolic blood pressure ≥ 90 mmHg. Childhood hypertension was determined by blood pressure values ≥ the modified sex- and age-specific criteria [3].

Blood was drawn from all study participants after they had fasted more than 8 hours. The levels of urine creatinine and serum creatinine, uric acid, low- and high-density lipoprotein cholesterol, triglyceride and glucose were measured with an autoanalyzer (Technician RA 2000 Autoanalyzer, Bayer Diagnostic, Mishawaka, IN) and are expressed as mg/dL. Serum insulin concentrations were quantified by the kit IMMULITE 2000 (Siemens Healthcare Diagnostics, Tarrytown, NY). The homeostasis model assessment of β-cell function (HOMA-β) and the homeostasis model assessment of insulin resistance index (HOMA-IR) were measured according to the models[4]. Serum adiponectin levels were measured by the kit Human Adiponectin/Acrp30 Immunoassay (R&D Systems, Minneapolis, MN). Diabetes mellitus was defined as a fasting serum glucose level ≥126 mg/dL or current use of medications to treat hyperglycemia.

***Measurements of urine metabolites of DEHP***

The detailed method has been introduced in our previous studies [5]. The urine samples were stored at −80°C before analysis. Standard DEHP metabolites and their corresponding isotopic ^13^C_4_-labeled compounds, were purchased from Cambridge Isotope Laboratories (Andover, MA, USA). Samples were then buffered with ammonium acetate [250 µL, 1M (pH 6.5)] and spiked with a mixture of 50 μL isotope internal standards (1 μg /mL) and beta-glucuronidase enzyme (20 µL, 200 Units/mL). Next, the sample was loaded into a solid phase extraction cartridge (Waters Oasis® HLB, 60 mg/3 mL, Hydrophilic-Lipophilic-Balanced). Aliquots of 3 mL each of 0.1% formic acid aqueous solution and 1 mL pure water were eluted to remove hydrophilic compounds. Then, 3 mL of methanol elution was added to collect the phthalate metabolites. The extract was dried with nitrogen gas and reconstituted with 0.1 mL of 20% methanol (aq) for analysis.

The chromatographic separation was performed using a Thermo Fisher-Accela UPLC system. The column was an Accucore C18 2.6 μm (2.1 × 100 mm) and was thermostatic at 25°C. The mobile phases, consisting of 5% acetonitrile aqueous solution with 0.1% acetic acid (Mobile phase A) and 0.1% acetic acid in acetonitrile (Mobile phase B), were delivered at a flow rate of 0.4 mL/min according to the following gradient: 0 min: (A)100%, 2 min: 80(A)/20(B)%, 17 min: 70(A)/30(B)%, 20 min: 20(A)/80(B)%, and 22 min: 80(A)/20(B)%. The column was re-equilibrated for 5 min. MS/MS detection was performed using a ThermoFisher-TSQ Quantum Access Triple Quadruple LC–MS/MS with an ESI source operating in the negative ion mode. The spray voltage was −3500 V, and the N_2_ sheath gas pressure was 45 psi. The N_2_ auxiliary gas pressure was 10 psi, the capillary temperature was 270°C, and the collision gas (Ar) pressure was 1.5 mTorr. The multiple reaction monitoring condition was used for isotope internal standard quantification. The total analysis time was 22 min for each sample. The limit of detection for the three urinary DEHP metabolites was 0.5 ng/mL. The pronounced R-squared of the calibration curve was higher than 0.995. The detection rate of DEHP is 78.4% among the study subjects and the limit of detection (LOD) is 0.5 ng/m. For concentrations below LOD, a value equal to the LOD divided by the square root of 2 was used.

***Analysis of leukocyte global DNA methylation levels***

The detailed method has been introduced in our previous studies [6]. The global DNA methylation level is usually expressed as [5mdC]/([dC] + [5mdC]). However, since the internal standard for each analyte requires custom synthesis [7], some studies expressed the global DNA methylation levels by 5mdC/dG based on the presumption dG = 5mdC + dC [8]; the use of dG as an internal standard is an accepted practice [6, 7]. In the current study, we measured 5mdC/dG with adjustment to the respective 15N-labeled internal standards and may be a more simple and accurate marker of dC methylation.

DNA extraction of global 5-methyl-2'-deoxycytidine (5-mdC) level genomic DNA was extracted from WBCs according to the procedures described [6]. Briefly, nuclease P1 was added to approximately 4 μg of genomic DNA and incubated at 37 °C for 2 h. Global 5-mdC levels were detected by high-performance liquid chromatography (Agilent 1260VL, Agilent Technology, U.S.A.) and introduced into a TurboIonSpray source using an ESI probe installed on an API 3000™ triple-quadrupole mass spectrometer (AB SCIEX. Canada). Data acquisition and quantitative processing were accomplished using Analyst 1.4.2™ software (AB SCIEX. Canada). The optimized source parameters multiple reaction monitoring mode transition pairs of dC, 5-mdC and ^15^N_3_-dC were set as m / z 228→112 m / z, m / z 242→126 m / z, and 231→115 for the quantitative pair, respectively.

Precision and accuracy were determined by analyzing DNA spiked with 5 ng / mL ^15^N_3_-dC (QC samples). The average intra-day and inter-day variations were 4.82% and 5.26% (n = 5), respectively. The accuracy was 95.43% (n = 5). QC samples and blank were measured after every 10 samples, and the calibration standard solution (middle concentration) was measured after every 20 samples. Our results were within acceptable limits and showed good reproducibility.

***ACE genotype analysis***

DNA was extracted from peripheral leukocytes of the subjects using standard methods. Genotyping of the *ACE* alleles (D/D, I/D, I/I) was performed using sequence-specific primer- polymerase chain reaction (PCR) methodologies[9, 10]. Primers were designed according to the ACE gene sequence and were synthesized by Invitrogen (Invitrogen Corporation, Carlsbad, CA, USA). The sequences of the forward and reverse primers 5′>CTG GAG ACC ACT CCC ATCCTTTCT>3′ and 5′>GAT GTG GCC ATC ACATTC GTC AGAT>3′, respectively. The reaction mixture was submitted to PCR amplification in a S1000 Thermal Cycler (Bio-Rad, Irvine, CA). A direct analysis of PCR product on a 1.8% agarose gel identified the three ACE I/D genotypes.

Supplemental table 1. Unstandardized regression coefficients (standard error) of cardiovascular disease risk factors with a one-unit increase in ln-DEHP metabolites (creatinine adjusted), serum adiponectin, and 5mdC/dG in multiple linear regression models

|  | n | SBP  mmHg | BMI z score  kg/m^2^ | LDL-C  mg/dL | HDL-C  mg/dL | ln-TG  mg/dL |
| --- | --- | --- | --- | --- | --- | --- |
| Ln-MEHP (μg/g creatinine) | 698 | 0.071 (0.182) | 0.049 (0.015) | 0.185 (0.451) | −0.178 (0.130) | -0.004 (0.006) |
| *P* value |  | 0.696 | 0.001 | 0.681 | 0.169 | 0.473 |
| Ln-MEHHP (μg/g creatinine) | 698 | -0.103 (0.468) | -0.019 (0.040) | −0.073 (1.158) | 0.226 (0.333) | 0.005 (0.016) |
| *P* value |  | 0.827 | 0.636 | 0.950 | 0.498 | 0.763 |
| Ln-MEOHP (μg/g creatinine) | 698 | 0.128 (0.481) | −0.022 (0.041) | −0.497 (1.188) | 0.155 (0.342) | −0.001 (0.016) |
| *P* value |  | 0.790 | 0.585 | 0.676 | 0.651 | 0.937 |
| Ln-5mdC/dG (%) | 612 | 0.446 (0.807) | 0.218 (0.070) | -0.590 (2.00) | 0.560 (0.576) | -0.053 (0.028) |
| *P* value |  | 0.581 | 0.002 | 0.768 | 0.331 | 0.057 |
| Ln-adiponectin (ng/ml) | 439 | -0.309 (0.613) | -0.309 (0.047) | -3.064 (1.397) | 2.188 (0.412) | -0.035 (0.020) |
| *P* value |  | 0.614 | <0.001 | 0.029 | <0.001 | 0.085 |

Data are means (standard error).

Adjusted for age, gender, BMI z score, smoking status, drinking status, and household income

Abbreviations: BMI z score, z score of body mass index; HOMA-IR, homeostasis model assessment of insulin resistance; LDL-C, low density lipoprotein cholesterol; MEHP, mono(2‐ethylhexyl) phthalate; MEHHP, mono(2‐ethyl‐5‐hydroxyhexyl) phthalate; MEOHP, mono(2‐ethyl‐5‐oxohexyl) phthalate; SBP, systolic blood pressure; TG, triglyceride.

#

Supplemental table 2. Mean (SE) of cardiovascular disease risk factors across ACE gene alleles in multiple linear regression models

|  | SBP  mmHg | BMI z score  kg/m^2^ | LDL-C  mg/dL | HDL-C  mg/dL | ln-TG  mg/dL | ln-HOMA-IR |
| --- | --- | --- | --- | --- | --- | --- |
| ACE gene phenotype |  |  |  |  |  |  |
| D/D | 106.91 (1.55) | 0.228 (0.13) | 102.14 (3.84) | 47.57 (1.10) | 4.31 (0.05) | -0.32 (0.11) |
| I/D | 108.05 (1.06) | 0.10 (0.09) | 102.20 (2.63) | 48.38 (0.75) | 4.34 (0.04) | -0.37 (0.07) |
| I/I | 108.48 (0.97) | -0.01 (0.08) | 104.02 (2.41) | 49.21 (0.69) | 4.31 (0.03) | -0.40 (0.07) |
| *P* for trend | 0.611 | 0.160 | 0.731 | 0.259 | 0.566 | 0.789 |

Data are means (standard error).

Adjusted for age, gender, BMI z score, smoking status, drinking status, and household income.

Abbreviations: BMI z score, z score of body mass index; HOMA-IR, homeostasis model assessment of insulin resistance; LDL-C, low density lipoprotein cholesterol; SBP, systolic blood pressure; TG, triglyceride.

**References**

1. Katsiki N, Mikhailidis DP, Gotzamani-Psarrakou A, Yovos JG, Karamitsos D: **Effect of various treatments on leptin, adiponectin, ghrelin and neuropeptide Y in patients with type 2 diabetes mellitus.** *Expert Opin Ther Targets* 2011, **15:**401-420.

2. **Global Database on Child Growth and Malnutrition** [<http://www.who.int/nutgrowthdb/software/en/>]

3. Battistoni A, Canichella F, Pignatelli G, Ferrucci A, Tocci G, Volpe M: **Hypertension in Young People: Epidemiology, Diagnostic Assessment and Therapeutic Approach.** *High Blood Press Cardiovasc Prev* 2015, **22:**381-388.

4. Wallace TM, Levy JC, Matthews DR: **Use and abuse of HOMA modeling.** *Diabetes care* 2004, **27:**1487-1495.

5. Lin CY, Hsieh CJ, Lo SC, Chen PC, Torng PL, Hu A, Sung FC, Su TC: **Positive association between concentration of phthalate metabolites in urine and microparticles in adolescents and young adults.** *Environ Int* 2016, **92-93:**157-164.

6. Liou SH, Wu WT, Liao HY, Chen CY, Tsai CY, Jung WT, Lee HL: **Global DNA methylation and oxidative stress biomarkers in workers exposed to metal oxide nanoparticles.** *J Hazard Mater* 2017, **331:**329-335.

7. Crain PF: **Preparation and enzymatic hydrolysis of DNA and RNA for mass spectrometry.** *Methods Enzymol* 1990, **193:**782-790.

8. Song L, James SR, Kazim L, Karpf AR: **Specific method for the determination of genomic DNA methylation by liquid chromatography-electrospray ionization tandem mass spectrometry.** *Anal Chem* 2005, **77:**504-510.

9. Chen S, Hao Q, Yang M, Yue J, Cao L, Liu G, Zou C, Ding X, Pu H, Dong B: **Association between Angiotensin-converting enzyme insertion/deletion polymorphisms and frailty among chinese older people.** *J Am Med Dir Assoc* 2015, **16:**438.e431-436.

10. Seripa D, Paroni G, Matera MG, Gravina C, Scarcelli C, Corritore M, D'Ambrosio LP, Urbano M, D'Onofrio G, Copetti M, et al: **Angiotensin-converting enzyme (ACE) genotypes and disability in hospitalized older patients.** *Age (Dordr)* 2011, **33:**409-419.
